# Differences in LC3B expression and prognostic implications in oropharyngeal and oral cavity squamous cell carcinoma patients

**DOI:** 10.1186/s12885-018-4536-x

**Published:** 2018-06-01

**Authors:** Kenneth Lai, Slade Matthews, James S. Wilmott, Murray C. Killingsworth, Jim L. Yong, Nicole J. Caixeiro, James Wykes, Allan Samakeh, Dion Forstner, Mark Lee, John McGuinness, Navin Niles, Angela Hong, Ardalan Ebrahimi, Cheok Soon Lee

**Affiliations:** 1 Sydney Medical School, The University of Sydney, Sydney, Australia; 2 Discipline of Pathology, School of Medicine, Western Sydney University, Sydney, Australia; 3 Centre for Oncology Education and Research Translation (CONCERT), Ingham Institute for Applied Medical Research, Sydney, Australia; 4 Department of Anatomical Pathology, Sydney South West Pathology Service (SSWPS) Liverpool Hospital, Sydney, Australia; 5 Bosch Institute, The University of Sydney, Sydney, Australia; 6 Melanoma Institute Australia, Sydney, Australia; 7 Faculty of Medicine, University of New South Wales, Sydney, Australia; 8 Department of Head & Neck Surgery, Liverpool Hospital, Sydney, Australia; 9 Department of Radiation Oncology, Liverpool Hospital, Sydney, Australia; 10 Ingham Institute for Applied Medical Research, 1 Campbell St, Liverpool, NSW 2170 Australia

**Keywords:** Autophagy, LC3B, Oropharyngeal, Oral cavity, SCC, HPV, Immunohistochemistry, Survival outcome

## Abstract

**Background:**

This study examined the prognostic significance of microtubule-associated protein light chain 3B (LC3B) expression in oropharyngeal and oral cavity squamous cell carcinoma (SCC). The prognostic significance of LC3B expression in relation to human papillomavirus (HPV) status in oropharyngeal SCC was also examined.

**Methods:**

Tissue microarrays (TMAs) were constructed from formalin-fixed, paraffin-embedded oropharyngeal (*n* = 47) and oral cavity (*n* = 95) SCC tissue blocks from patients with long-term recurrence and overall survival data (median = 47 months). LC3B expression on tumour was assessed by immunohistochemistry and evaluated for associations with clinicopathological variables. LC3B expression was stratified into high and low expression cohorts using ROC curves with Manhattan distance minimisation, followed by Kaplan–Meier and multivariable survival analyses. Interaction terms between HPV status and LC3B expression in oropharyngeal SCC patients were also examined by joint-effects and stratified analyses.

**Results:**

Kaplan–Meier survival and univariate analyses revealed that high LC3B expression was correlated with poor overall survival in oropharyngeal SCC patients (*p* = 0.007 and HR = 3.18, 95% CI 1.31–7.71, *p* = 0.01 respectively). High LC3B expression was also an independent prognostic factor for poor overall survival in oropharyngeal SCC patients (HR = 4.02, 95% CI 1.38–11.47, *p* = 0.011). In contrast, in oral cavity SCC, only disease-free survival remained statistically significant after univariate analysis (HR = 2.36, 95% CI 1.19–4.67, *p* = 0.014), although Kaplan-Meier survival analysis showed that high LC3B expression correlated with poor overall and disease-free survival (*p* = 0.046 and 0.011 respectively). Furthermore, oropharyngeal SCC patients with HPV-negative/high LC3B expression were correlated with poor overall survival in both joint-effects and stratified presentations (*p* = 0.024 and 0.032 respectively).

**Conclusions:**

High LC3B expression correlates with poor prognosis in oropharyngeal and oral cavity SCC, which highlights the importance of autophagy in these malignancies. High LC3B expression appears to be an independent prognostic marker for oropharyngeal SCC but not for oral cavity SCC patients. The difference in the prognostic significance of LC3B between oropharyngeal and oral cavity SCCs further supports the biological differences between these malignancies. The possibility that oropharyngeal SCC patients with negative HPV status and high LC3B expression were at particular risk of a poor outcome warrants further investigation in prospective studies with larger numbers.

## Background

Oropharyngeal and oral cavity squamous cell carcinoma (SCC) make up the majority of head and neck cancers and combined, rank as the eighth most common cancer worldwide [1, 2]. The incidence of oropharyngeal SCC has increased substantially in developed countries over the past few decades while the incidence of oral cavity SCC has remained stable or even decreased [3–10]. Although oropharyngeal and oral cavity SCC are often collectively studied as “oral SCC” as well as aggregated with other head and neck cancers, these malignancies are distinctively different from one another including the impact of human papillomavirus (HPV) infection, biology and treatment approaches [11]. HPV positive oropharyngeal SCC patients tend to display a better survival outcome in comparison to HPV negative patients [12–14]. In contrast, the clinical significance of HPV infection in oral cavity SCC is ambiguous. [15–23].

Macroautophagy (referred to as autophagy hereafter) is a process of cellular self-consumption for recycling of intracellular components and has recently received much interest in cancer therapeutic research due to its unique role in both pro- and anti-cancer activity [24]. Autophagy begins with the formation of a phagophore that can be either generated by de novo formation or from various cellular components including the plasma membrane, Golgi apparatus, endoplasmic reticulum and outer mitochondrial membrane [25]. During autophagy induction, intracellular components are sequestered by phagophores and develop into autophagosomes that fuse with lysosomes, mature into autolysosomes for degradation and generate into amino acids for biomass and/or energy production [24]. Autophagy can be either non-selectively targeting cytoplasm for bulk degradation or selectively targeting cellular components including aggregated proteins and damaged organelles [26]. Autophagy helps to maintain cellular homoeostasis but it can also be upregulated in response to various stresses including pathogen invasion, cytotoxicity, oxygen and nutrient deprivation [26]. Furthermore, imbalance of autophagy is associated with numerous diseases such as systemic lupus erythematosus, Crohn’s disease and cancers [27].

The role of autophagy in cancer progression remains controversial due to its possession of both pro- and anti-cancer properties [28]. On the pro-cancer side, autophagy provides amino acids as an alternative energy source for cancer cell proliferation as well as generates resistance toward radiotherapy and chemotherapy. On the other hand, autophagy can also lead to type II programmed cell death [24]. Regarding therapeutic uses, autophagy inhibitors are shown to increase the potency of various chemotherapy agents in cancers. Clinical trials investigating the effectiveness of autophagy inhibitors in combination with immunotherapy, targeted therapy, and chemotherapy in cancers have been launched since 2010 and display encouraging preliminary results [29]. Recently, nanomedicine that involves a polymeric co-delivery system, allowing the sequential release of the autophagy inhibitor, LY294002, and a chemotherapeutic agent, doxorubicin, displayed promising results in the oral cavity (tongue) SCC cell lines [30].

Microtubule-associated protein light chains 3 (LC3) is a specific autophagosome marker and has been demonstrated to be an effective prognostic marker in various cancers including oral SCC [31, 32]. LC3 participates in autophagosome membrane elongation, and its activated form binds tightly to the pre-autophagosomal, autophagosomal and autolysosomal membranes [24, 33, 34]. LC3 consists of three main members, which include LC3A, LC3B and LC3C [35]. Increased expression in LC3A and LC3B correlates with poor prognosis in various cancers including breast cancer, colorectal cancer, gastric cancer and oral SCC [31, 32, 36–40]. LC3C is lesser known, and its prognostic value in cancer remains unclear. Although high LC3B expression has been associated with poor disease-free survival in oral SCC patients [32], some studies incorrectly regard oral SCC as both oropharyngeal and oral cavity SCC [11]. As oropharyngeal and oral cavity SCC are distinctive SCC subgroups due to their different biology and management [11], a more definitive LC3B prognostic assessment between these malignancies would help to further establish their association with autophagy activity and thus assess the effectiveness of utilizing autophagy as a therapeutic strategy in oropharyngeal and oral cavity SCC.

The present study further examines for any difference in LC3B expression between oropharyngeal and oral cavity SCC patients through LC3B immunohistochemistry assessment and correlation with prognosis, clinical and pathological characteristics of patients. The combined effects of HPV and LC3B expression as predictors of outcome in oropharyngeal SCC patients were also examined.

## Methods

### Study cohorts

Patients with T1–4, N0–3, M0 diagnosed between 2000 and 2014 were identified from the database of the NSW Cancer Registry. Department of Anatomical Pathology databases, hospital and surgeon records were used to verify and input missing data as required. Retrieved data was validated by the treating clinicians (JW and AS). Patients with missing and/or incomplete follow up, and treatment records were excluded from the study. Patients were followed up for the occurrence of an event, which was defined as recurrence in any form or death from any cause, for between 2 and 275 (median = 47) months after diagnosis. A total of 142 oropharyngeal (n = 47) and oral cavity (*n* = 95) SCC patients were included in the study. The formalin fixed paraffin embedded (FFPE) tissue of the primary tumors, as well as their corresponding hematoxylin and eosin stained (H&E) slides were obtained from the Department of Anatomical Pathology, Liverpool Hospital, New South Wales, Australia.

### Tissue microarray (TMA) construction

H&E slides were examined by light microscopy and located regions of interest (ROI) including the central and peripheral regions of the tumor as well as lymph node metastases where applicable. Using the H&E slides as a reference, duplicate tissue cores from each ROI were removed from the FFPE tissue blocks (donor blocks) and inserted into a blank paraffin block (recipient block) using MTA-1 manual tissue arrayer (Beecher Instruments, Sun Prairie, USA). All TMA blocks were sectioned at 3 μm thickness and collected on Superfrost plus glass slides (Thermo Fisher Scientific, Waltham, USA) before immunohistochemistry.

### Immunohistochemistry

LC3B immunohistochemistry (IHC) staining was performed manually. All procedures were performed at room temperature unless otherwise specified. All involved reagents were manufactured by Dako, Glostrup, Denmark. Sections were rinsed with EnVision FLEX Wash Buffer after each incubation step until the antibody binding visualisation. Sections were deparaffinised in xylene and rehydrated through graded alcohol. Heat induced antigen retrieval was carried out using EnVision FLEX Target Retrieval Solution High pH for 20 min at 98 °C. Endogenous peroxidase was quenched in all sections with Dual Endogenous Enzyme Block for 10 min. Sections were incubated with mouse monoclonal antibodies against LC3B (1:50, clone 5F10, NanoTools, Teningen, Germany) for 90 min then incubated with HRP conjugated secondary antibody for 30 min. Antibody binding was visualised by incubating with Liquid DAB+ Substrate Chromogen System for 5 min. Sections were counterstained with Harris haematoxylin and Scott’s bluing reagent, dehydrated with alcohol and xylene, and mounted on glass coverslips. IHC staining on tumor section without the primary antibody was performed as the negative control.

### Evaluation of immunohistochemistry

The intensity and percentage of the LC3B expression in each tissue sample were evaluated semi-quantitatively by four independent pathologists, including a senior pathologist (CSL). The scorers underwent a period of training with a multiheaded microscope to ensure consistent and reliable interpretation. Using a test series of at least 36 tissue core sections, intra- and inter-observer agreement was estimated using Kappa (κ) and Spearman rho (ρ). Training was ended when the desired level of agreement, consistent over time, was achieved (κ > 0.6 and ρ > 0.8). An average score was obtained from the duplicate cores of each tissue sample. All researchers were blinded to clinical and other laboratory data. LC3B expression was presented as cytoplasmic punctate staining. The intensity of LC3B expression was graded as follow: 0 (≤10 punctate staining per cell), 1 (11–20 punctate staining per cell), 2 (> 20 punctate staining per cell without clustering) and 3 (> 20 punctate staining per cell with clustering) while the percentage of LC3B positive tumor cells was recorded from 0 to > 75%. The results of staining were calculated using a quick (Q) score, which was achieved by multiplying the percentage of positive cells (P) by the intensity (I) hence the final score ranged from 0 to 225 [41]. LC3B expression in oropharyngeal and oral cavity SCCs were stratified using Budczies et al.’s Cutoff Finder application, which employed ROC curve analysis with the Manhattan distance minimization approach to threshold optimization predicting death from the LC3B value [42].

### Statistical analyses

Clinicopathological characteristics of oropharyngeal and oral cavity SCCs, as well as associations between LC3B expression cohorts and clinicopathological characteristics in both SCC types, were assessed using a two-sample t-test for the continuous variables and chi-squared or Fisher’s exact tests for categorical variables. The parameters were dichotomised where possible to assist the analyses.

Survival analyses were conducted for the outcomes of both overall and disease-free survival, with time to each outcome calculated from the date of diagnosis. Disease-free survival describes the period of time patients spend free of remission of disease and is the cumulative figure derived from all patients. Overall survival is calculated the same way but with an outcome measure of mortality rather than remission. All survival analyses were performed using IBM SPSS Statistic software version 22 (IBM, New York, USA). An event was defined as recurrence in any form or death from any cause, with only the first event taken into account. Patients without events were censored at the date of last known follow-up. Data were right-censored only. Unadjusted survival curves were obtained using Kaplan Meier estimates and compared with Log-Rank test. Cox proportional-hazards models were used to estimate the hazard ratio of clinicopathological characteristics and LC3B expression about both overall and disease-free survival in oropharyngeal and oral cavity SCCs separately. Interaction terms between HPV status and LC3B expression in oropharyngeal SCC patients were used to assess whether HPV modified the effect of LC3B expression on survival when examined in joint-effects and stratified analyses. Results for all analyses were only considered to be statistically significant if the associated *p*-value was less than 0.05.

## Results

### LC3B immunohistochemical staining pattern on tumor cells

**Fig. 1:**
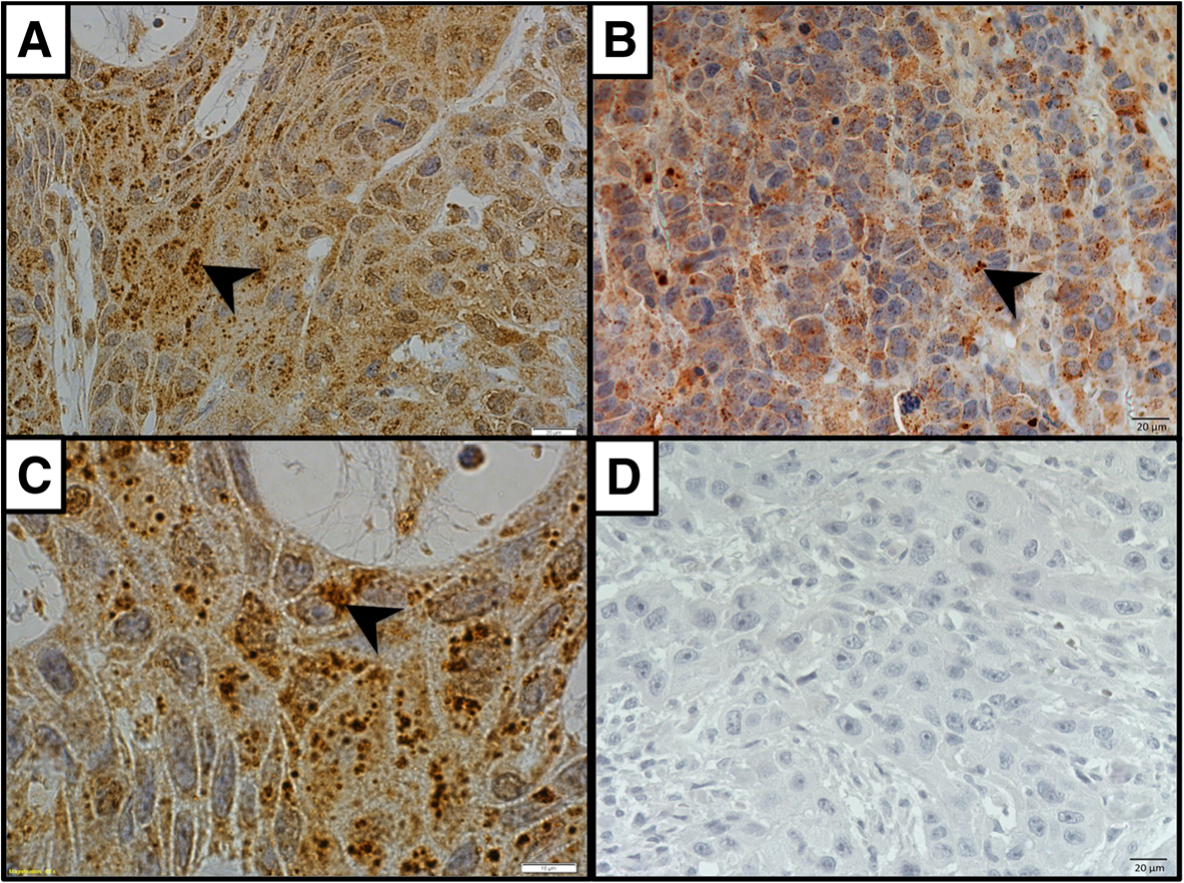
LC3B immunohistochemical staining pattern. A&B. LC3B (clone 5F10) expression appeared as cytoplasmic punctate (black arrowheads) in both oropharyngeal (**a**) and oral cavity (**b**) SCC cells. **c** Cytoplasmic punctate LC3B staining under higher magnification (× 100). **d** Negative control (performed immunohistochemical staining without LC3B antibody incubation). Magnification of A, B and D: × 40, C: × 100

LC3B expression appeared as cytoplasmic punctate staining in both oropharyngeal and oral cavity SCC cells (Fig. 1). No distinctive expressional difference was observed between oropharyngeal and oral cavity SCC cells. The intensity of the punctate staining pattern appeared to be heterogeneous in tumor cells (Fig. 1). Limited LC3B punctate staining was also observed in normal epithelial cells.

### Differential expression of LC3B about demographic and clinical characteristics between oropharyngeal and oral cavity SCC patients

LC3B expression was stratified into low and high based on ROC curves with Manhattan distance minimization to perform the survival-data-based cut-off determination [42]. LC3B Q scores above 140.6 were stratified as high and predictive of death by the algorithm in both oropharyngeal and oral cavity SCC. Differences in LC3B expression about demographic and clinical characteristics between oropharyngeal and oral cavity SCC patients were compared and summarized in Table 1. Oropharyngeal SCC patients comprised approximately one-third of the study population (34%) while the median age at diagnosis was identical in both SCC types (60 years). High LC3B expression was observed in 45% of oropharyngeal SCC whereas only 29% of oral cavity SCC had a high LC3B expression (*p* = < 0.0001). There was no significant difference in demographic or clinical characteristics in oropharyngeal SCC patients with different LC3B expression. On the contrary, oral cavity SCC patients with high LC3B expression were more likely to develop the recurrent disease compared to patients with low LC3B expression (54 and 28% respectively, *p* = 0.02).

**Table 1:** LC3B expressions and clinicopathologic variables in oropharyngeal and oral cavity SCC patients

	Oropharyngeal SCC				Oral Cavity SCC			
	Total	Low LC3B	High LC3B	*P* value	Total	Low LC3B	High LC3B	*P* value
Total	47	26 (55%)	21 (45%)	< 0.0001	95	67 (71%)	28 (29%)	< 0.0001
Age	41–83 (60)	43–83 (59)	41–80 (62)	0.46	28–97 (60)	28–97 (62)	34–83 (58)	0.21
≤ 60 years	27	18 (69%)	9 (43%)	0.07	50	33 (49%)	17 (61%)	0.31
> 60 years	20	8 (31%)	12 (57%)		45	34 (51%)	11 (39%)	
Gender								
Male	36	19 (73%)	17 (81%)	0.53	60	41 (61%)	19 (68%)	0.54
Female	11	7 (27%)	4 (19%)		35	26 (39%)	9 (32%)	
Tumour stage								
1 & 2	26	18 (69%)	8 (38%)	0.6	67	46 (69%)	21 (75%)	0.54
3 & 4	21	8 (31%)	13 (62%)		28	21 (31%)	7 (25%)	
Nodal stage								
0 & 1	22	11 (42%)	11 (52%)	0.49	71	53 (79%)	18 (64%)	0.13
2 & 3	25	15 (58%)	10 (48%)		24	14 (21%)	10 (36%)	
TNM stage								
I & II	10	6 (23%)	4 (19%)	0.74	47	34 (51%)	13 (46%)	0.7
III & IV	37	20 (77%)	17 (81%)		48	33 (49%)	15 (54%)	
Tumour grade	missing = 3				missing = 2			
1 & 2	28	15 (60%)	13 (68%)	0.57	75	55 (85%)	20 (71%)	0.14
3	16	10 (40%)	6 (32%)		18	10 (15%)	8 (29%)	
Recurrence								
Absent	30	18 (69%)	12 (57%)	0.39	61	48 (72%)	13 (46%)	0.02*
Present	17	8 (31%)	9 (43%)		34	19 (28%)	15 (54%)	
Smoking status	missing = 2				missing = 9			
Never-smoker	3	2 (67%)	1 (33%)	1.00^a^	20	15 (75%)	5 (25%)	0.78^a^
Ex & current smoker	42	23 (55%)	19 (45%)		66	46 (70%)	20 (30%)	

^a^= Fisher’s exact test is performed due to one of the cell frequency is less than or equals to 5

*= statistical significance (*p* < 0.05)

### Kaplan Meier survival analysis

**Fig. 2:**
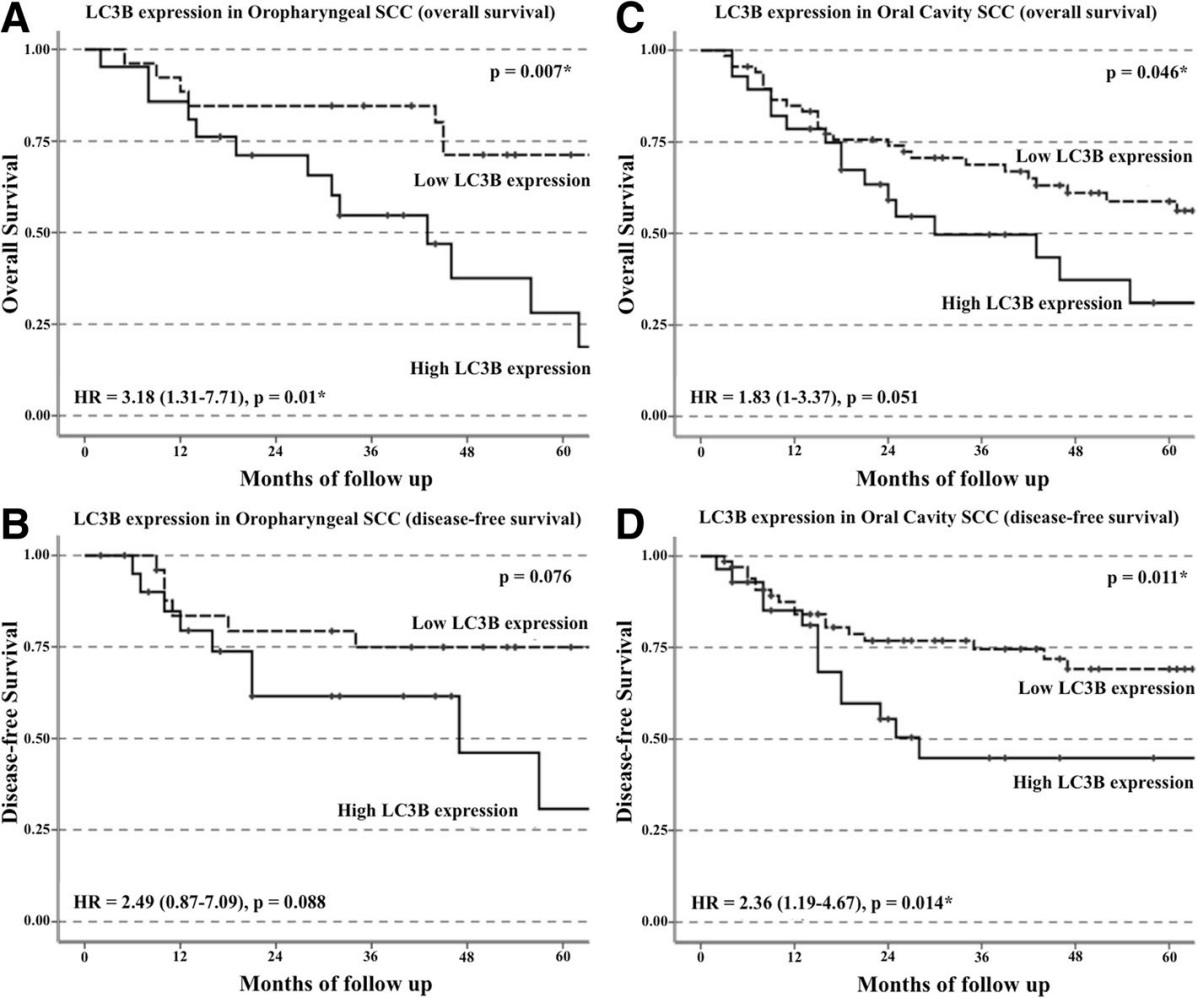
Kaplan Meier survival curves of LC3B expression in oropharyngeal (*n* = 47) and oral cavity (*n* = 95) SCC patients. Although oropharyngeal SCC patients with high LC3B expression displayed worse survival than patients with low LC3B expression in both overall survival (**a**) and disease-free survival (**b**), only overall survival reached statistical significance (*p* = 0.007, Log Rank test). Oral cavity SCC patients with high LC3B expression displayed worse survival than patients with low LC3B expression in both overall survival (**c**) and disease-free survival (**d**) (*p* = 0.046 and 0.011 respectively, Log Rank test)

Differences in LC3B expression about overall and disease-free survival in oropharyngeal and oral cavity SCCs were determined by Kaplan Meier survival and univariate Cox regression analyses (Fig. 2).

Oropharyngeal SCC patients with high levels of LC3B expression displayed significantly worse overall survival than patients with low levels of LC3B expression (Fig. 2a). At five years, the overall survival of patients with high levels of LC3B expression was approximately 30% while patients with low levels of LC3B expression remained around 70% (*p* = 0.007). Furthermore, patients with high levels of LC3B expression also displayed a high hazard ratio under univariate Cox regression analysis (HR = 3.18, 95% CI 1.31–7.71, *p* = 0.01). There was a trend towards worse disease-free survival in patients with high LC3B expression (Fig. 2b). However this failed to reach statistical significance (*p* = 0.076).

In oral cavity SCC patients, Kaplan Meier survival analysis showed that patients with high levels of LC3B expression had significantly worse overall and disease-free survival than patients with low levels of LC3B expression (Fig. 2c & d). The overall survival of patients (Fig. 2c) with high levels of LC3B expression decreased to approximately 30% while patients with low levels of LC3B was double (60%) at five years (*p* = 0.046). Regarding disease-free survival (Fig. 2d), patients with high levels of LC3B expression decreased to approximately 45% while patients with low levels of LC3B expression remained at 70% at five years (*p* = 0.011). Univariate Cox regression analysis revealed that oral cavity SCC patients with high levels of LC3B expression displayed a higher hazard ratio in disease-free survival (HR = 2.36, 95% CI 1.19–4.67, *p* = 0.014), although a relatively high ratio was also observed in the overall survival, it is just short of statistical significance (HR = 1.83, 95% CI 1–3.37, *p* = 0.051).

### Cox proportional hazard ratio analysis

The prognostic significance of LC3B expression in oropharyngeal and oral cavity SCC patients was further analysed using univariate and multivariate Cox proportional hazard ratio models, as summarized in Tables 2 and 3 respectively.

**Table 2:** Univariate and multivariate Cox proportional hazard analyses of clinicopathologic variables for overall and disease-free survival in oropharyngeal SCC patients

Oropharyngeal SCC					
Variable	Univariate analysis			Multivariate analysis	
	*Hazard ratio (95% Cl)*	*P value*		*Hazard ratio (95% Cl)*	*P value*
2A. Overall survival					
*Age* (> 60 yr. vs ≤ 60 yr)	2.13 (0.96–4.72)	0.062		2.55 (0.86–7.58)	0.093
*Gender* (male vs female)	0.64 (0.25–1.69)	0.372		0.23 (0.07–0.76)	0.016*
*Smoking status* ^a^ (ex & current vs never)	1.49 (0.2–11.23)	0.697		0.64 (0.07–5.68)	0.691
*Tumour grade* ^b^ (3 vs 1&2)	0.69 (0.27–1.81)	0.456		0.23 (0.07–0.78)	0.018*
*T stage* (3&4 vs 1&2)	1.51 (0.69–3.31)	0.307		0.27 (0.07–1.03)	0.055
*N stage* (2&3 vs 0&1)	1.39 (0.64–3.05)	0.407		5.21 (1.43–19.01)	0.012*
*LC3B expression* (high vs low)	3.18 (1.31–7.71)	0.01*		4.02 (1.38–11.74)	0.011*
2B. Disease-free survival					
*Age* (> 60 yr. vs ≤ 60 yr)	3.9 (1.35–11.26)	0.012*		3.24 (0.89–11.81)	0.075
*Gender* (male vs female)	0.75 (0.24–2.39)	0.631		0.5 (0.11–2.35)	0.379
*Smoking status* ^a^ (ex & current vs never)	NC	NC		NC	NC
*Tumour grade* ^b^ (3 vs 1&2)	0.61 (0.19–1.98)	0.409		0.39 (0.09–1.78)	0.226
*T stage* (3&4 vs 1&2)	1.46 (0.55–3.91)	0.447		0.53 (0.13–2.14)	0.372
*N stage* (2&3 vs 0&1)	1.21 (0.45–3.26)	0.705		2.33 (0.57–9.49)	0.237
*LC3B expression* (high vs low)	2.49 (0.87–7.09)	0.088		1.91 (0.57–6.36)	0.293

^a^= missing 2 cases

^b^= missing 4 cases

*= statistical significance (*p* < 0.05)

NC = not calculated (coefficient cannot be estimated due to imbalance data)

**Table 3:** Univariate and multivariate Cox proportional hazard analyses of clinicopathologic variables for overall and disease-free survival in oral cavity SCC patients

Oral cavity SCC				
Variable	Univariate analysis		Multivariate analysis	
	*Hazard ratio (95% Cl)*	*P value*	*Hazard ratio (95% Cl)*	*P value*
3A. Overall survival				
*Age* (> 60 yr. vs ≤ 60 yr)	2.25 (1.24–4.11)	0.008*	3.01 (1.38–6.56)	0.006*
*Gender* (male vs female)	1.02 (0.55–1.88)	0.949	0.89 (0.43–1.84)	0.748
*Smoking status* ^a^ (ex & current vs never)	0.56 (0.28–1.13)	0.104	0.37 (0.17–0.82)	0.014*
*Tumour grade* ^b^ (3 vs 1&2)	2.32 (1.24–4.32)	0.008*	2.29 (1–5.26)	0.051
*T stage* (3&4 vs 1&2)	1.68 (0.92–3.09)	0.093	1.49 (0.7–3.16)	0.301
*N stage* (2&3 vs 0&1)	1.46 (0.78–2.73)	0.243	1.97 (0.77–5.05)	0.158
*LC3B expression* (high vs low)	1.83 (1–3.37)	0.051	1.67 (0.78–3.58)	0.19
3B. Disease-free survival				
*Age* (> 60 yr. vs ≤ 60 yr)	1.69 (0.86–3.35)	0.13	1.49 (0.63–3.49)	0.361
*Gender* (male vs female)	0.9 (0.45–1.8)	0.767	0.77 (0.33–1.82)	0.553
*Smoking status* ^a^ (ex & current vs never)	0.93 (0.38–2.27)	0.867	0.68 (0.26–1.82)	0.446
*Tumour grade* ^b^ (3 vs 1&2)	1.82 (0.87–3.83)	0.114	2.16 (0.79–5.91)	0.132
*T stage* (3&4 vs 1&2)	1.09 (0.51–2.36)	0.82	0.81 (0.33–2)	0.643
*N stage* (2&3 vs 0&1)	2.07 (1.02–4.22)	0.045*	3.42 (1.21–9.67)	0.021*
*LC3B expression* (high vs low)	2.36 (1.19–4.67)	0.014*	1.82 (0.83–4)	0.138

^**a**^= missing 9 cases

^**b**^= missing 2 cases

*= statistical significance (*p* < 0.05)

Clinicopathological features that are associated with better overall survival of patients with oropharyngeal SCC include male gender, and ironically with advanced tumor grade (Table 2) under multi-variable analysis (HR = 0.23, 95% CI 0.07–0.76, *p* = 0.016 and HR = 0.23, 95% CI 0.07–0.78, *p* = 0.018 respectively), but the latter is most likely related to HPV status. As previously mentioned, HPV positive oropharyngeal SCC patients tend to display better survival outcome than HPV-negative patients while such population is rapidly increased in male patients [12]. Furthermore, HPV positive SCC cells often displayed basaloid differentiation that is considered as advanced grade [43]. On the other hand, advanced N stages were associated with higher risk under multi-variable analysis (HR = 5.21, 95% CI 1.43–19.01, *p* = 0.012). In the case of LC3B expression, patients with high LC3B expression were exposed to higher risk under both univariate and multivariate analysis (HR = 3.18, 95% CI 1.31–7.71, *p* = 0.01 and HR = 4.02, 95% CI 1.38–11.74, *p* = 0.011 respectively), the significance of the biomarker is retained when the influence of other parameters is accounted by the multivariate analysis suggesting it is an independent prognostic marker.

Considering overall survival of oral cavity SCC patients (Table 3A), age greater than 60 years was associated with higher risk under both univariate and multivariate analysis (HR = 2.25, 95% CI 1.24–4.11, *p* = 0.008 and HR = 3.01, 95% CI 1.38–6.56, *p* = 0.006 respectively). Although univariate analysis of patients with advanced tumor grade was also shown to be at higher risk (HR = 2.32, 95% CI 1.24–4.32, *p* = 0.008), the influence of this variable was reduced when other covariates were introduced into the model using multivariate analysis (HR = 2.29, 95% CI 1–5.26, *p* = 0.051). In the disease-free survival of oral cavity patients (Table 3B), patients with advanced N stages were associated with higher risk under both univariate and multivariable analysis (HR = 2.07, 95% CI 1.02–4.22, *p* = 0.045 and HR = 3.42, 95% CI 1.21–9.67, *p* = 0.021 respectively). However, high LC3B expression did not appear to be an independent prognostic factor in either overall or disease-free survival for the oral cavity SCC patients (*p* = 0.19 and 0.138 respectively). Smoking status appeared to introduce a systematic bias into the multivariable analysis precluding the construction of a stable model as reported in our previous study [14].

### Effect of combining HPV and LC3B in oropharyngeal SCC patients

HPV is known to be an important prognostic factor in oropharyngeal SCC patients as HPV-positive patients tend to display better prognosis [12, 13], similarly, our previous study with the same cohort had also observed that HPV positivity is associated with better survival outcome in oropharyngeal SCC but not in oral cavity SCC patients [14]. To further investigate whether HPV modified the effect of LC3B expression on the survival of oropharyngeal SCC patients, the prognostic significance of a combination of HPV and LC3B expression was evaluated in joint-effects and stratified analyses, as summarized in Table 4. After adjustment for age, gender, tumor grade, T- and N-stage, the best outcomes were seen in patients with HPV-positive/low LC3B expression cancers and the worst in those with HPV-negative/high LC3B expression cancers. Relative to patients with HPV-positive/low LC3B expression cancers, those with HPV-negative/high LC3B expression cancers displayed poor overall survival in the joint-effects analyses (HR = 4.76, 95% CI 1.23–18.48, *p* = 0.024). This effect was particularly pronounced in the HPV negative patients as revealed by the stratified presentation (HR = 18.71, 95% CI 1.3–270.24, *p* = 0.032). Although a similar trend was also observed in the disease-free survival, it was not statistically significant (*p* > 0.05).

**Table 4:** Association between HPV and LC3B status on overall and disease-free survival in oropharyngeal SCC (*n* = 47)

LC3B and HPV status	Overall survival		Disease-free survival	
	HR* (95% CI)	*P value*	HR* (95% CI)	*P value*
A. Joint-effects presentation				
HPV positive/Low LC3B (*n* = 18)	1.00		1.00	
HPV positive/High LC3B (*n* = 11)	2.85 (0.77–10.47)	0.115	1.91 (0.36–10.22)	0.449
HPV negative/Low LC3B (*n* = 8)	0.82 (0.24–2.82)	0.756	1.45 (0.30–7.15)	0.647
HPV negative/High LC3B (*n* = 10)	4.76 (1.23–18.48)	0.024*	2.83 (0.57–0.57)	0.205
B. Stratified presentation				
HPV positive/Low LC3B (*n* = 18)	1.00		1.00	
HPV positive/High LC3B (*n* = 11)	2.95 (0.66–13.25)	0.159	1.83 (0.28–11.79)	0.524
HPV negative/Low LC3B (*n* = 8)	1.00		1.00	
HPV negative/High LC3B (*n* = 10)	18.71 (1.3–270.24)	0.032*	3.35 (0.31–36.78)	0.323

Clinical variables adjusted

*= statistical significance (*p* < 0.05)

## Discussion

In the current study, the LC3B expression on immunohistochemistry is characterized by a punctate cytoplasmic pattern in both oropharyneal and oral cavity SCC cells. To date, LC3B expression patterns in cancer are reported predominantly cytoplasmic [38, 39, 44–50], meanwhile other patterns such as large globule (stone) like structure and crescentic (perinuclear) patterns are also observed in oesophageal adenocarcinoma and triple negative breast cancer (TNBC) [39, 48]. In the case of oral SCC, the LC3B expression is characterized by punctate cytoplasmic pattern [32], which is consistent with the current study.

High LC3B expression correlates with poor prognosis in both oropharyngeal and oral cavity SCC with stronger prognostic significance found in oropharyngeal SCC patients. Oropharyngeal SCC patients with HPV-negative/high LC3B expression were found to have poorer overall survival. LC3B is reported to be an effective prognostic marker in various cancers. The high LC3B expression is an independent prognostic marker for poor overall and disease-free survival in locally advanced breast cancer and TNBC [38, 39]. In astrocytoma, high LC3B expression alone, as well as high co-expression with CD133, a cancer stem cell-like marker, is associated with poor overall survival [46]. In the case of hepatocellular carcinoma, the high LC3B expression is associated with advanced TNM stages, vascular invasion, lymph node metastasis as well as an independent prognostic marker for poor overall survival [47]. In prostate adenocarcinoma, the high LC3B expression is an independent prognostic marker for a high Gleason score [45]. Despite that multiple LC3B expression patterns are observed in oesophageal adenocarcinoma, only large globule like structure pattern emerged as an independent prognostic marker for poor overall survival irrespective of treatment [48]. In the case of oral SCC, although Kaplan Meier and univariate analyses show that high LC3B expression correlated with poor disease-free survival, it did not appear to be an independent prognostic factor in multivariate analysis [32]. The current study further investigated for any prognostic difference in LC3B expression between oral cavity and oropharyngeal SCC. Similar to Liu et al., high LC3B expression is associated with poor overall and disease-free survival in oral cavity SCC, but it also did not emerge as an independent prognostic marker. In contrast to oral cavity SCC, the high LC3B expression is strongly associated with poor overall survival outcome in oropharyngeal SCC patients thus raising the possibility of its use as an independent prognostic marker. Our previous study showed that HPV is associated with better survival outcome in oropharyngeal SCC but not in oral cavity SCC patients [14], furthermore, the current study also showed that patients with HPV-negative/high LC3B expression displayed the most unfavourable survival outcome. Since LC3B displays different prognostic value amongst different cancers, it is likely that our finding further supports the biological differences between oropharyngeal and oral cavity SCC.

The cohort was not analysed based on different treatment regimes because of the small number of cases, which resulted in numbers that are too small in the stratified groups to have any meaningful statistical power. However, in future an expanded cohort will be required to generate data that has the sufficient statistical power be analysed in the context of the recently updated OPSCC nomogram [51].

Although the current study demonstrated that high autophagy correlates with poor prognosis in oropharyngeal and oral cavity SCC, autophagy can be involved in either the promotion or inhibition of cancer cell survival. Atg6/Beclin-1 is thought to suppress tumorigenesis, meanwhile, damage-regulated autophagy modulator (DRAM) is essential for p53 mediated apoptosis and p53 also induces autophagy in a DRAM-dependent manner [24].

It is essential to further clarify the role of autophagy in oropharyngeal and oral cavity SCC progression as such information would become useful when autophagy is considered within therapeutic strategies for these tumors. Inhibition of autophagy through pharmacological inhibitors and RNA interference (RNAi) of autophagy-related genes is shown to enhance chemosensitivity and photosensitivity in cancer cell models [24]. Specifically, an in-vitro study showed that depletion of LC3 gene using RNAi enhances the sensitivity of hepatocellular carcinoma cells to Epirubicin [52]. 3-Methyladenine (3-MA), which inhibits autophagy by preventing autophagosome formation via the inhibition of class III phosphatidylinositol 3-kinase (PI3K), is shown to enhance the cytotoxicity of numerous chemotherapy agents including Cisplatin, 5-fluorouracil (5-FU), Tamoxifen, Trastuzumab and Camptothecin [53–58]. Similar to 3-MA, Chloroquine (CQ), a 4-aminoquinoline drug that is widely used to treat malaria, prevents autolysosome fusion, and it is also reported to enhance the efficacy of Cisplatin, 5-FU, Gefitinib and Paclitaxel [59–65]. Radiotherapy (RT) is reported to induce autophagy activity in cancer cells and speculated to play a major role in RT resistance. Autophagy inhibition through CQ and/or RNAi increases the radiosensitivity and chemo-radiosensitivity in cancer cell lines including breast carcinoma, colorectal cancer, non-small cell lung cancer and glioma stem cells [66–69]. Although the therapeutic significance of autophagy inhibition in response to RT is yet to be functionally tested on oropharyngeal and oral cavity SCC cells, autophagy activity is reported to be elevated in irradiated oral cavity SCC cells [70]. Recently, the trial of a nanomedicine employing a polymeric co-delivery system, allowing the sequential release of the autophagy inhibitor, LY294002, and a chemotherapeutic agent, doxorubicin, shows promising results in oral cavity (tongue) SCC cells [30]. As the current study observed that autophagy is associated with both oropharyngeal and oral cavity SCC progression, future studies should compare the effects of autophagy inhibition between oropharyngeal and oral cavity SCC cell lines in response to radio and chemotherapy. Additionally, combined investigation between LC3B expressions and other independent prognostic markers including EGFR status, matted nodes, p27 and cyclin D1 in oropharyngeal SCC patients might help to further stratify other patient subgroups for different therapeutic approaches [71–73].

## Conclusions

In conclusion, we propose that LC3B is an independent prognostic marker for oropharyngeal SCC patients due to the strong association between high LC3B expression and poor overall survival outcome in our patient cohort; however, this was not observed in oral cavity SCC patients. Furthermore, the current study further supports a biological difference between oropharyngeal and oral cavity SCC as LC3B expression displayed a different prognostic significance in these malignancies. As autophagy appears to be involved in oropharyngeal and oral cavity SCC progression, future studies should evaluate the effects of autophagy inhibition of these tumors in response to chemo radiotherapy and chemotherapy. The possibility that oropharyngeal SCC patients with negative HPV status and high LC3B expression were at particular risk of a poor outcome warrants further investigation in prospective studies with larger numbers. If our findings are confirmed, pretreatment testing for LC3B expression in addition to HPV will help to better stratify oropharyngeal SCC patients in the setting of tailored treatment. In particular, the group of patients with HPV-negative/high LC3B expression cancers would benefit from intensified treatment.

## Abbreviations

3-MA: 3-Methyladenine
5-FU: 5-fluorouracil
CQ: Chloroquine
FFPE: Formalin fixed paraffin embedded
HPV: Human papillomavirus
I: Intensity
IHC: Immunohistochemistry
LC3: Microtubule-associated protein light chain 3
P: Positive cells
PI3K: Class III phosphatidylinositol 3-kinase
Q: Quick score
RNAi: RNA interference
ROI: Regions of interest
RT: Radiotherapy
SCC: Squamous cell carcinoma
TMAs: Tissue microarrays
TNBC: Triple negative breast cancer
